# Reciprocal Associations between Depressive Symptoms, Life Satisfaction, and Eudaimonic Well-Being in Older Adults over a 16-Year Period

**DOI:** 10.3390/ijerph20032374

**Published:** 2023-01-29

**Authors:** Mohsen Joshanloo, Ana Blasco-Belled

**Affiliations:** 1 Department of Psychology, Keimyung University, Daegu 42601, Republic of Korea; 2 Department of Psychology, University of Girona, Plaça Sant Domènec, 9, 17004 Girona, Spain

**Keywords:** depressive symptoms, life satisfaction, eudaimonic well-being, purpose in life, within-person, RI-CLPM, ELSA

## Abstract

The dual-continua model of mental health distinguishes between mental illness (presence of mental disorders, such as depression) and mental well-being (presence of positive traits and abilities). This model also distinguishes between hedonic well-being (e.g., affect balance and life satisfaction) and eudaimonic well-being (i.e., optimal psychological and social functioning, as indicated for example by having a purpose in life). We examined the relationships between depressive symptoms (a common indicator of mental illness), life satisfaction, and eudaimonic well-being. The study used a sample of 17,056 participants from England whose data were collected at eight intervals of approximately two years over a 16-year period, from 2004 to 2019. The mean age of the sample in the first wave was 58.843 years, with a standard deviation of 12.617 years (women = 55.2%). We disentangled within- and between-person sources of variance to examine whether increases or decreases in one variable preceded changes in the other variables at the next time point. We found positive reciprocal relationships between life satisfaction and eudaimonic well-being and negative reciprocal relationships between the two well-being dimensions and depressive symptoms. These results suggest that within-person increases in well-being are followed by future decreases in depressive symptoms, and within-person increases in depressive symptoms are followed by future decreases in well-being. Therefore, low levels of mental well-being in older adults may be considered a risk factor for depression, and well-being interventions (such as those focused on meaning-making) may serve as a protective factor against depression in older adults.

## 1. Introduction

Promoting mental health is a top priority in psychology. This requires identifying the components that define mental health and their correlates, and developing effective interventions tailored to contextual and individual factors. The traditional view of mental health as consisting solely of the absence of mental illness has been replaced by models that understand mental health as a multidimensional construct that also includes positive attributes. Mental health is now primarily understood as a system of complex relationships between indicators of well-being and ill-being [1]. The dual-continua model of mental health [2] recognizes this complexity and is consistent with the World Health Organization’s view that mental health is more than the absence of psychopathology [3]. This model assumes that mental health consists of two independent but interrelated continua: mental well-being and mental illness [2,4]. In this framework, mental health is defined by high levels of mental well-being and low levels of mental illness. This view implies that increases in mental well-being are not necessarily associated with decreases in mental illness, and that treatment of psychopathology is not necessarily associated with improvements in positive well-being. It is possible to experience mental well-being even when mental illness is present [5,6], just as low well-being is not synonymous with psychopathology [7].

The dual-continua model of mental health combines two predominant conceptualizations used to describe the defining characteristics of mental well-being: hedonic and eudaimonic well-being [8]. According to the dual-continua model of mental health, hedonic and eudaimonic components are both necessary and complementary elements of mental well-being. Hedonic well-being (also known as subjective well-being) includes affective and cognitive components. Affective well-being consists of the presence of pleasant feelings (e.g., feeling happy and cheerful) and the absence of unpleasant feelings (e.g., feeling depressed and anxious). Life satisfaction is the cognitive component of subjective well-being that refers to a person’s overall evaluation of his or her life [9].

Eudaimonic well-being, in contrast, refers to the realization of human potential, the pursuit of meaningful activities, and purposeful living [10,11]. Eudaimonic well-being comprises unique aspects of positive functioning that are not part of hedonic well-being, such as relational skills or meaning in life [12,13]. The concept of eudaimonia comes from classical philosophy. To cast it into a concept that can be defined and measured, psychologists have defined eudaimonia “as an active striving toward virtuous excellence and perfection” ([14], p. 178). The psychological models of eudaimonic well-being emphasize different types of virtuous striving, such as meaning-making, personal growth, or self-enrichment [15]. For example, Ryff’s [10] model of psychological well-being is a widely used conceptualization of eudaimonic well-being [16]. This model includes self-acceptance, positive relationships, autonomy, personal growth, purpose in life, and environmental mastery [17]. Some of these elements are also recognized in self-determination theory [18] as the central components of eudaimonic well-being: autonomy, competence, and relatedness. The common components of eudaimonic well-being across these models depend, to varying degrees, on having a direction and a coherent system of meaning for one’s life. After all, the pursuit of excellence and perfection depends on having a fairly clear idea of what excellence and perfection mean in the context of one’s life. This demonstrates the importance of living a meaningful and purposeful life in order to achieve eudaimonic well-being.

### 1.1. Interplay between Mental Well-Being and Illness

The distinction between mental well-being and mental illness makes it possible to examine the complicated interactions between the two. A common mental disorder that has received much attention is depression, a multidimensional mental disorder characterized by anhedonia and psychosocial dysfunction [19]. It is one of the leading causes of disability worldwide and poses a major challenge to the global healthcare system [20,21]. Rates of this debilitating disorder continue to increase [22]. Approximately 240 million people worldwide suffer from depression, with just under 40% achieving full recovery [23]. Therefore, identifying and improving the mechanisms involved in the initiation and maintenance of depression is of primary interest [21]. Despite improvements in the treatment of depression, the prevalence of this debilitating disorder does not appear to have decreased, raising important questions about understanding, prevention, and treatment [24] and opening new avenues for advances in the field [25].

Within the dual-continua framework, researchers have examined whether mental well-being prevents people from becoming mentally ill. Studies show that depression and mental well-being are negatively correlated. For example, one study found that individuals with low mental well-being had more than seven times the risk of developing depression 10 years later, and twice the risk when demographic, personality, and health-related variables were taken into account [26]. Studies also show that improved mental well-being is generally protective against mental illness [27,28,29,30,31].

### 1.2. Limitations of Prior Research

Research on the interactions between mental well-being and depression is characterized by some limitations: 1- Most of the prior studies in this area have used samples of young and middle-aged individuals (e.g., [30,32,33,34,35], while few studies have analyzed these associations in older adults (e.g., [26,29]). 2- Extant research generally does not examine the reciprocal associations between mental well-being and depression, but consider one as a predictor and the other as an outcome (e.g., [36,37]). 3- Even rarer are studies that assess within-person associations. Extant research generally has looked at between-person associations and has not considered how variations within individuals in one variable predict within-person variations in the other variable. There are exceptions, of course. An example is an experience-sampling study in which 25 students were intensively observed for two weeks. The study found a moderate negative relationship between mental well-being and depression at the within-person level [38]. Another example is a study that examined the within-person relationships between life satisfaction and depression [28], and found that life satisfaction predicted depression up to six years later, whereas depression predicted life satisfaction only up to three years later. Yet, the latter study had a notable drawback: it only measured hedonic well-being, leaving unanswered the question of how within-person changes in eudaimonic well-being and depressive symptoms are related. 

### 1.3. Mental Health in Older Adulthood

The transition from middle to older adulthood is characterized by many personal and social challenges such as retirement or health problems, which can potentially lead to a decline in mental well-being and an increase in depressive symptoms [17,39]. Meaning and purpose in life (i.e., eudaimonic well-being) contribute to healthier aging and are related to fewer depressive symptoms in old age [40]. On this basis, eudaimonic interventions are effective in promoting hedonic and eudaimonic well-being and reducing depressive symptoms in middle and older adulthood [41,42].

Understanding the longitudinal interactions between mental well-being and depressive symptoms in adults could reveal mechanisms associated with better longevity and mental health. To this end, we need to understand the reciprocal inter-relations as dynamic processes that span over different life stages [43,44]. Longitudinal studies are most appropriate because this research design examines psychological phenomena as they unfold in a naturalistic setting, especially when methodological approaches that separate between- and within-person effects are used [45]. Such research may provide insights not only into the protective role of mental well-being against depression over time but also into the circumstances in which psychological interventions might be effective for healthier, more meaningful aging [44].

### 1.4. The Present Study

Most previous studies in this area have measured outcomes and predictors on a single occasion. Cross-sectional data do not lend themselves well to the study of processes that are considered dynamic. This is because cross-sectional data make it difficult to discern the dynamics of individual change needed to rule out alternative explanations. Accordingly, analyses based on cross-sectional data can lead to an incomplete understanding of processes and misleading interpretations [46]. Time is a key factor in understanding how developmental processes unfold and how their effects can be observed, and thus longitudinal data are critical for progress in this area.

The present study aimed to use longitudinal data to examine the temporal within-person associations between depressive symptoms, life satisfaction, and eudaimonic well-being. Life satisfaction is usually considered a component of hedonic well-being. Therefore, in this study, both hedonic and eudaimonic well-being are measured along with depressive symptoms, which serve as a general indicator of mental illness. A large sample from the British general population was used, collected at eight time points approximately two years apart over approximately 16 years. Given these characteristics of the sample, this study aimed to provide insight into the longitudinal relationships between these variables over long periods of time and in the general population.

The Random-Intercept Cross-Lagged Panel Model (RI-CLPM; [47]) was used to disentangle sources of variation between and within individuals. Our main focus is on the within-person (intra-individual) links between the three variables. We define within-person change as a deviation from the typical level of a variable for an individual. The RI-CLPM can answer the question of whether a change in one variable is associated with a future change in another variable [48] after accounting for the temporal stability of the variables.

## 2. Methods

### 2.1. Participants

The data used in this study are from the English Longitudinal Study of Aging (ELSA). The ELSA samples individuals that are living in private households in England and are aged 50 years or older. The initial ELSA sample was drawn from participants in three different years of the Health Survey for England (1998, 1999, and 2001). These years were selected because they offered a substantial sample size and were the most recent at the time. The core samples from these years were nationally representative (https://www.elsa-project.ac.uk/study-documentation, accessed on 1 October 2022). In fact, an examination of the ELSA participant’s demographic information in comparison to national census data revealed that the ELSA sample closely mirrored the composition of the general population in England [49]. New participants of younger age have been added to the sample at regular intervals to ensure that it remains representative of the entire age spectrum [50,51]. The first wave of ELSA took place in 2002/2003, and individuals have been resurveyed every two years since then. Currently, nine waves of ELSA data are available. The ninth wave of data collection took place in 2018–2019. This study used data from eight waves (Waves 2–9), given that life satisfaction was not measured in the first wave. These ELSA waves are referred to as time points 1 to 8 in the present study. From a total of 19,807 participants, 2751 (13.9%) did not answer any of the 3 variables of this study and were thus excluded. Except for a complete lack of data on study variables, no other participant was excluded for any other reason. The sample used consisted of 17,056 participants who responded to at least one of the scales in one of the eight time points (mean age at the first time point = 58.843, median = 58, SD = 12.617, women = 55.2%, mean age at the last time point = 73.843, median = 73).

### 2.2. Data Availability

All data and survey materials are publicly available. More information can be found at https://www.elsa-project.ac.uk/accessing-elsa-data (accessed on 1 October 2022).

### 2.3. Measures

The Cronbach’s alpha coefficients and descriptive information for the three variables at the first time point are shown in Table 1. The alpha coefficients and descriptive information for other time points are shown in Table S1.

Life satisfaction. Life satisfaction was evaluated with the Satisfaction with Life Scale (SWLS; [52]). The scale has 5 items, each rated on a 7-point scale (1 = strongly disagree to 7 = strongly agree). Principal axis factoring results using the five items at time point 1 were consistent with a single-factor structure. The first initial eigenvalue was 3.629 and the second was 0.546. The single factor explained 66.371% of the total variance in the responses. Factor loadings ranged between 0.646 and 0.908.

Eudaimonic well-being. We chose 9 items from the 19-item version of the control, autonomy, self-realization, and pleasure scale (CASP-19; [53]) for the measurement of eudaimonic well-being. The items used in this study assess attitudes related to eudaimonic well-being as outlined in the models of Ryff [10] and Ryan and Deci [8], which include aspects such as self-acceptance, personal growth, environmental mastery, autonomy, and a sense of purpose in life. The selected items are presented in the Appendix A. The CASP-19 items are rated on a scale ranging between 1 = often and 4 = never. The items were recoded wherever needed such that higher scores indicated higher levels of eudaimonic well-being. Our initial inspection of the items of this scale indicated that the scale consisted of a broad range of items tapping into various domains of quality of life. We chose only items that matched existing models of eudaimonic well-being [8,10]. We also relied on a list of eudaimonic variables to identify the components of eudaimonic well-being [54], such as competence, meaning, engagement, optimism, and vitality, among others.

Principal axis factoring with the nine items at time point 1 showed that a one-factor model was consistent with the data. The first initial eigenvalue was 3.913, the second was 1.038, and the third was 0.892. The single factor explained 37.012% of the total variance in the responses. Factor loadings ranged between 0.468 and 0.809.

Depressive symptoms. The ELSA uses an 8-item version of the Center for Epidemiologic Studies Depression Scale (CES-D; [55]), a widely used self-assessment of depressive symptoms used in population-based studies to identify individuals at risk for depression. Respondents indicated whether they experienced the eight symptoms much of the time during the past week, using a yes/no response format. Responses to two positive items (“happy” and “enjoyed life”) were reverse-coded so that a “no” response to these questions was considered an additional symptom. A score was calculated for each participant ranging from 0 (no self-reported symptom) to 8 (eight self-reported symptoms). Principal axis factoring with the eight items at time point 1 showed that a one-factor model was consistent with the data. The first initial eigenvalue was 3.409, the second was 1.001, and the third was 0.808. The single factor explained 34.862% of the total variance in the responses. Factor loadings ranged between 0.366 and 0.728.

### 2.4. Statistical Analysis

Model estimation and fit evaluation. Models were estimated using Robust Maximum Likelihood (MLR) under missing data theory in Mplus version 8.8. A Comparative Fit Index (CFI) value of >0.95, a Root Mean Square Error of Approximation (RMSEA) value of <0.07, and a Standardized Root Mean Square Residual (SRMR) value of <0.08 were used as thresholds for a good model fit in this study (e.g., [56]).

RI-CLPM. An RI-CLPM was simultaneously tested for all three variables. The variables were included as observed variables. Baseline age and gender were included as time-invariant covariates of the observed variables across time points. In accordance with common practices in the RI-CLPM [57], the auto-regressive and cross-lagged effects were constrained to equality over time to obtain omnibus tests of associations between the variables.

Missing data handling. Only participants who did not respond to any of the study measures across the eight time points were excluded from this study. Of the included participants, 18.0% participated at all 8 time points, and 82.0% of participants had at least 1 missing wave (i.e., did not respond to any of the variables in a wave). Participants with missing waves were not excluded from the analysis because the current study used Full Information Maximum Likelihood to use all available data. A longitudinal missing data indicator was calculated with values ranging from 0 (no missing wave) to 7 (seven missing waves). The results show that there were weak correlations between the missing data indicator and the main variables of the study at time point 1 (Table 1). The correlations for other time points are shown in Table S2. These results suggest a general tendency for individuals with more missing waves to have poorer mental health, albeit with a very small effect size. To account for this pattern, the missing data indicator was included as an auxiliary variable in the model. The auxiliary variables are not part of the main model. They are included because they are related to missingness or variables with missing data. The inclusion of auxiliary variables can reduce the bias in parameters that can result from attrition, thereby improving the accuracy of parameter estimates [56,58].

## 3. Results

The intercorrelations between the variables for time point 1 are shown in Table 2. Table 3 shows the correlations between the central variables of the study for time point 1 and 8. The intercorrelations for the variables across the eight time points are shown in Table S2.

An RI-CLPM with the three variables was tested. The model fit the data very well (Chi-Square value = 977.659, degree of freedom = 237, *p* < 0.001, RMSEA = 0.014, 90% confidence interval for RMSEA = 0.013–0.014, CFI = 0.993, SRMR = 0.039). The parameter estimates of interest are presented in Table 4. As the correlations between the trait components show, the variables are strongly related at the between-person level. However, the between-person correlations do not tell us much about how the variables are related over time. They predominantly reflect synchronicity and/or common causal influences. The within-person level results provide information about the temporal relationships between changes in the variables. The autoregressive effects are all positive and significant, indicating that a change in one variable is associated with a change in the same variable and in the same direction at the next time point. Thus, if an individual has a higher value than his or her typical level of one variable, he or she is also more likely to have a higher value on the variable at the next time point. It is expected and typically found in panel data that increases or decreases in a variable persist to some extent across time.

All of the cross-lagged effects were also significant, indicating that within-person increases or decreases in one variable are associated with within-person increases or decreases in the other variables at the next time point. In other words, deviations in one variable are associated with future deviations in the other two variables. All the within-person associations are mutual and significant. Life satisfaction and eudaimonic well-being have positive within-person associations, whereas the within-person association between depressive symptoms and the well-being variables is negative. Based on the guidelines provided by [59], the within-person association between the two well-being variables is moderate to strong, and all other associations involving depressive symptoms (as an outcome or predictor) can be considered weak to moderate effects.

## 4. Discussion

Disentangling the interactions between mental well-being and mental illness requires not only consideration of their conceptual complexities, similarities, and differences, but also a refined understanding of their temporal relations. To this end, we analyzed the within-person associations between life satisfaction, eudaimonic well-being, and depressive symptoms in a sample of older adults over 16 years. In our RI-CLPM, we disaggregated within- and between-person sources of variance to directly examine whether individual deviations from the mean values of one variable preceded changes in another variable at the next time point. We expected that an increase in depressive symptoms would be a precursor to lower life satisfaction and eudaimonic well-being over time. Well-being variables were also expected to predict future reductions in depressive symptoms. The results were in line with our expectations. We found positive reciprocal relationships between life satisfaction and eudaimonic well-being, and negative reciprocal relationships between the two well-being dimensions and depressive symptoms.

Our study overcomes some common drawbacks in the literature, including the dearth of studies examining variation in mental health at the within-person level and in older adulthood. While most longitudinal studies examining the interplay between mental well-being and mental illness have provided a solid background for understanding differences across individuals [26,29,31,33], progress in this field also requires understanding what characterizes these differences at the within-person level; that is, how these variables interact after ruling out between-person differences. Below, we explain the results at the within- and between-person levels.

### Interplay between Mental Well-Being and Depressive Symptoms

To untangle the temporal interplay between the indicators of mental well-being and mental illness, it is crucial to control for the temporally stable variance of the variables. This is an important prerequisite if we are to elucidate the mechanisms responsible for the specific longitudinal interactions between these variables. Accordingly, we separated within- and between-person sources of variance in our model. The time-invariant part of the variance for each variable (the trait component) contains the temporally stable part of the variance in each variable. The correlations between these trait components do not capture longitudinal changes and are not temporal.

We examined the within-person associations between variables after accounting for the stable between-person variance. These time-varying components reflect changes from the expected level of the variable at each time point. We looked at the cross-lagged effects between these time-varying components. The cross-lagged within-person effects showed that an increase in depressive symptoms was followed by a decrease in mental well-being, whereas an increase in mental well-being was followed by a decrease in depressive symptoms over time. That is, time-specific deviations from average mental well-being and depressive symptoms predicted fluctuations in the other variables at the next time point. These within-person results suggest that an adult who exhibits higher-than-usual mental well-being at a point in time is likely to report fewer depressive symptoms after two years. While the magnitudes of reciprocal effects between life satisfaction and eudaimonic well-being were similar, these two indicators showed slightly stronger effects in predicting depressive symptoms than the other way around. This could be interpreted as an indication that mental well-being in older adulthood seems to be a better predictor of mental illness than the other way around.

Considering variance decomposition is critical to evaluating the contribution of our study, as failure to separate within- and between-person sources of variation can not only inflate lagged paths but also lead to misleading conclusions about the relations between variables [57,60]. For example, when variance is not partitioned (as in a traditional CLPM), lagged effects reflect a puzzling mix of within- and between-person associations among variables, and pure within-person effects are not identifiable. Mere reliance on methods that do not partition variance (such as the traditional CLPM) may lead to inaccurate conclusions about the causal processes that characterize the complex interplay between mental well-being and mental illness.

Studies of the relationships between mental well-being and illness are scarce, and to our knowledge, only a few studies [28,38], have examined these relationships at the within-person level. These studies have found reciprocal associations between the variables. Our results extend and complement the previous studies by showing that mental well-being (including both hedonic and eudaimonic well-being) and depressive symptoms have reciprocal relationships. These results show that even when hedonic and eudaimonic well-being are considered simultaneously, both components uniquely predict future depressive symptoms at the within-person level. Thus, neither component is redundant, and both deserve due attention in prevention efforts.

In terms of between-person effects, our findings align with previous studies highlighting the protective role of mental well-being against mental illness [27,31], and the potential risk of low mental well-being for developing psychological symptoms [26,33]. The between-person findings indicate that, on average, individuals with higher mental well-being report less mental distress, and vice versa. But directionality cannot be inferred from between-person results. Our study, by contrast, disentangled the within- and between-person level variance to rule out trait stability in the variables and inspect only within-person covariance to draw directional conclusions. Thus, our mutual within-person effects are not because some people tend to report high/low levels of both mental well-being and depressive symptoms. Instead, these effects suggest that decreases or increases in the variables are associated with decreases/increases in future levels of the other variables.

## 5. Implications

The present results extend findings from cross-sectional between-person studies and suggest that reciprocal associations between indicators of mental well-being and mental illness also exist at the within-person level, contributing to our understanding of the causal processes of mental health in older adulthood. The fact that the pathways from life satisfaction and eudaimonic well-being to depressive symptoms were somewhat stronger than the pathways from depressive symptoms to well-being variables suggests that positive mental health may indeed be a particularly important protective factor against future mental illness in older adults. If deterioration in mental well-being is a precursor to depressive symptoms, it may be possible to detect early signals of deterioration in mental well-being and take steps to prevent the negative consequences. Similarly, promoting mental well-being could be seen as a worthwhile investment to avoid the detrimental effects of depressive symptoms. This may be particularly important in order to mitigate vulnerabilities associated with the transition to older adulthood. Engagement in hedonic and eudaimonic well-being appears to be instrumental in facilitating healthier, more meaningful aging.

There is some evidence that eudaimonia-based group interventions can improve mental well-being and reduce psychological distress (including anxiety and depressive symptoms) in older adults [61,62]. The idea that different types of psychological interventions can help improve psychological well-being is supported by meta-analytic findings [63]. In these interventions, participants are typically taught strategies to identify and promote well-being in their daily lives, particularly with regard to areas that may improve (e.g., self-acceptance) or deteriorate (e.g., purpose in life) as a result of normative life transitions. Serrano et al. [41] and Friedman et al. [42] found that benefits were more pronounced for individuals with lower baseline levels of well-being. Multicomponent positive psychological interventions that include hedonic (e.g., positive emotions) and eudaimonic (e.g., meaning in life) elements have also been shown to be effective in improving mental well-being and alleviating psychological symptoms in older adults [64,65]. Other interventions, including activities focused on autobiographical memories, forgiveness, gratitude [66], self-compassion [67], life review therapy [68], and prevention of social isolation [69] have also demonstrated mental health benefits in adults over age 60.

Eudaimonic well-being plays an important role across one’s lifespan, not only because it is associated with fewer symptoms of mental illness but also because it helps individuals cope with age-related challenges [70]. Eudaimonic well-being interventions would particularly benefit from incorporating purpose and meaning-enhancing activities [71]. Purposeful engagement is associated with improvements in physical health, including reduced probability for all-cause mortality or Alzheimer’s disease, among others (see [72] for a review). Practitioners should therefore adapt purpose-in-life activities, considering how the conceptualization of this construct changes across the lifespan, so that older adults find value and engagement in their daily hobbies, social interactions, or routines [73].

One of the reasons why the effects of depressive symptoms may be exacerbated in older adults is that older adults may have difficulty performing routine daily activities [74]. An additional challenge is that certain components of eudaimonic well-being, such as purpose in life or personal growth, tend to decline at this stage of life [39,75]. Westerhof and Keyes [4] found that older adults did not exhibit better mental well-being compared to younger adults, although they reported fewer symptoms of mental illness. This may be due to a lack of adequate opportunities for the development of meaning and purpose in older adulthood [76]. Our findings underscore the need to promote mental well-being at this stage of life. In particular, meaning-based eudaimonic interventions should be an important element of prevention efforts in these age groups. Interventions that focus on age-appropriate purposeful and meaningful activities are needed as a prevention strategy to ensure mental health and reduce healthcare costs [77].

## 6. Limitations

Our study is not without limitations. We used a sample of older English adults, so generalization to groups with other demographic characteristics and from different national contexts requires further investigation. In addition, we used a general population sample, and the applicability of our results to any clinical context is subject to clinician discretion. Because of the lack of experimental control, causal inferences should be avoided. Associations between mental well-being and mental illness could be due to unmeasured (e.g., biological or environmental) variables. We used a composite measure of eudaimonic well-being in this study because the CASP items we used could not be statistically grouped into sufficiently distinct factors. That is, this study was unable to provide a robust multidimensional assessment of eudaimonic well-being and instead assessed it as a general concept that encompasses eudaimonic items across different components. It is also worth noting that the CASP is not specifically designed as a measurement tool for eudaimonic well-being. For future research, it is important to further investigate the different components of eudaimonic well-being and their unique relationship to the development of depressive symptoms. Different facets of eudaimonic well-being such as meaning in life, personal growth, environmental mastery, and self-acceptance [10] should be distinguished to improve our understanding of the bidirectional and intrapersonal relationships between different aspects of well-being and depression. A more detailed and multidimensional understanding of eudaimonic well-being is crucial because it is needed to develop eudaimonic interventions to reduce the risk of depressive symptoms. Thus, various components of eudaimonia, particularly those that decline with age, such as purpose and growth [39,75], should be the focus of future studies with aging populations.

It is also important to recognize that the cultural context in which mental well-being [78] and mental illness [79] are experienced can significantly influence these experiences. A cross-cultural comparative approach would involve comparing the results of this study to similar studies conducted in other cultural contexts to determine if the associations found in this study are consistent or differ across cultures. It would be imperative to consider how cultural factors such as collectivism or individualism, lay conceptions of emotions, well-being, aging, and various religious and spiritual beliefs may influence the experience of mental well-being and its association with symptoms of mental disorders across cultures. For example, research has shown that East Asians tend to be more tolerant of negative emotions than Westerners due to their holistic thinking style, which views negative emotions as malleable and transient [80]. Similarly, Muslims may show greater tolerance of negative emotions than Westerners because, in Islamic cultures, adversity and difficulties are viewed as tests or trials of faith to be endured with gratitude and trust in God. Islamic scriptures recognize various divine purposes for suffering, including discipline and spiritual growth [81]. Given such cultural differences, the findings of this study cannot be generalized to other cultures without empirical investigation. There is a lack of within-person research in this area worldwide, and more research is needed across cultures. This could lead to a more nuanced understanding of the factors that contribute to the mental well-being of older adults and allow for the development of culturally sensitive models and interventions.

## 7. Conclusions

In summary, our study brings a much-needed perspective to the research field by examining the within-person relationships between depressive symptoms, life satisfaction, and eudaimonic well-being. The results of the study offer new insights into the directionality of these relationships and can be used to inform the development of mental well-being interventions. Although the study had some limitations, such as the failure to account for the complexity of eudaimonic well-being, the findings nevertheless represent a valuable contribution to mental health research. Our study highlights the importance of understanding the complex intraindividual relationships between different aspects of mental well-being and mental illness. The findings also highlight the need for further research with diverse populations and a more comprehensive approach to understanding eudaimonic well-being. Future studies should aim to replicate and extend the findings of this study to gain a more robust understanding of the relationships between these variables.

## Figures and Tables

**Table 1:** Descriptive Statistics and Internal Reliabilities (Time Point 1).

	Eudaimonic	Life Satisfaction	Depressive
Valid	8271	8270	9220
Missing	11,536	11,537	10,587
Mean	3.206	5.259	1.575
Std. Deviation	0.554	1.234	1.959
Skewness	−0.918	−1.068	1.448
Std. Error of Skewness	0.027	0.027	0.026
Kurtosis	0.649	0.822	1.389
Std. Error of Kurtosis	0.054	0.054	0.051
Minimum	1.000	1.000	0.000
Maximum	4.000	7.000	8.000
Cronbach’s alpha	0.827	0.896	0.789

**Table 2:** Correlation Matrix.

	Eudaimonic	Satisfaction	Depressive	Age	Gender
Eudaimonic	1				
Life satisfaction	0.618 ***	1			
Depressive	−0.514 ***	−0.446 ***	1		
Age	0.143 ***	−0.035 **	−0.079 ***	1	
Female	−0.004	−0.015	0.127 ***	0.029 ***	1
Number of missing waves	−0.169 ***	−0.038 **	0.106 ***	−0.001	−0.044 ***

Note. *** *p* < 0.001. ** *p* < 0.01. Eudaimonic well-being, life satisfaction, and depressive symptoms in this table were measured at time point 1. The gender variable is coded as 0 = male, 1 = female.

**Table 3:** Correlation Matrix for Main Variables for Time Points 1 and 8.

	Eudaimonic 1	Satisfaction 1	Depressive 1	Eudaimonic 8	Satisfaction 8
Eudaimonic 1	--				
Life satisfaction 1	0.618	--			
Depressive 1	−0.514	−0.446	--		
Eudaimonic 8	0.503	0.398	−0.267	--	
Life satisfaction 8	0.401	0.533	−0.268	0.648	--
Depressive 8	−0.356	−0.278	0.369	−0.533	−0.458

Note. All correlations are significant at *p* < 0.001. Eudaimonic well-being, life satisfaction, and depressive symptoms in this table were measured at time points 1 and 8.

**Table 4:** Parameter Estimates for RI-CLPM.

Predictor	Outcome	Unstandardized Coefficient	*p*	95% CI		Standardized Coefficient
				Low	Up	
Auto-regressive						
Eudaimonic	Eudaimonic	0.184	0.000	0.165	0.204	0.208
Life satisfaction	Life satisfaction	0.200	0.000	0.180	0.219	0.183
Depressive	Depressive	0.126	0.000	0.110	0.143	0.128
Cross-lagged						
Life satisfaction	Eudaimonic	0.039	0.000	0.033	0.046	0.097
Depressive	Eudaimonic	−0.011	0.000	−0.014	−0.008	−0.051
Depressive	Life satisfaction	−0.033	0.000	−0.041	−0.025	−0.055
Eudaimonic	Life satisfaction	0.248	0.000	0.209	0.287	0.104
Eudaimonic	Depressive	−0.238	0.000	−0.296	−0.180	−0.061
Life satisfaction	Depressive	−0.093	0.000	−0.118	−0.068	−0.051
Trait covariances						
Eudaimonic	Life satisfaction	0.365	0.000	0.352	0.378	0.785
Life satisfaction	Depressive	−0.907	0.000	−0.950	−0.864	−0.649
Depressive	Eudaimonic	−0.487	0.000	−0.507	−0.467	−0.775
State covariances (time point 1)						
Eudaimonic	Life satisfaction	0.132	0.000	0.119	0.145	0.448
Life satisfaction	Depressive	−0.327	0.000	−0.374	−0.279	−0.280
Depressive	Eudaimonic	−0.158	0.000	−0.177	−0.138	−0.295
State covariances (time point 2)						
Eudaimonic	Life satisfaction	0.130	0.000	0.119	0.140	0.497
Life satisfaction	Depressive	−0.311	0.000	−0.352	−0.269	−0.261
Depressive	Eudaimonic	−0.109	0.000	−0.124	−0.094	−0.247

Note. CI = confidence interval. All regressive paths are held equal across waves. The standardized regression coefficients are related to the paths between time points 1 and 2. Regression coefficients related to other waves are similar to the reported coefficients and are not reported here due to space limitations. State covariances related to other waves are similar to the reported ones and are not reported here.

## Data Availability

The data used in this study are publicly available at https://www.elsa-project.ac.uk/accessing-elsa-data (accessed on 1 October 2022).

## Supplementary Materials

The following supporting information can be downloaded at: https://www.mdpi.com/article/10.3390/ijerph20032374/s1, Table S1: Descriptive Statistics; Table S2: Intercorrelations.

## Appendix A

The CASP items we used in this study to measure eudaimonic well-being.

1. I feel that what happens to me is out of my control
2. I feel free to plan for the future
3. I feel left out of things
4. I can do the things I want to do
5. I look forward to each day
6. I feel that my life has meaning
7. I choose to do things that I have never done before
8. I feel that life is full of opportunities
9. I feel that the future looks good to me
